# Collaboration between CpG sites is needed for stable somatic inheritance of DNA methylation states

**DOI:** 10.1093/nar/gkt1235

**Published:** 2013-11-27

**Authors:** Jan O. Haerter, Cecilia Lövkvist, Ian B. Dodd, Kim Sneppen

**Affiliations:** ^1^Center for Models of Life, Niels Bohr Institute, University of Copenhagen, Blegdamsvej 17, DK-2100 Copenhagen, Denmark and ^2^Department of Molecular and Biomedical Sciences (Biochemistry), University of Adelaide, SA 5005, Australia

## Abstract

Inheritance of 5-methyl cytosine modification of CpG (CG/CG) DNA sequences is needed to maintain early developmental decisions in vertebrates. The standard inheritance model treats CpGs as independent, with methylated CpGs maintained by efficient methylation of hemimethylated CpGs produced after DNA replication, and unmethylated CpGs maintained by an absence of *de novo* methylation. By stochastic simulations of CpG islands over multiple cell cycles and systematic sampling of reaction parameters, we show that the standard model is inconsistent with many experimental observations. In contrast, dynamic collaboration between CpGs can provide strong error-tolerant somatic inheritance of both hypermethylated and hypomethylated states of a cluster of CpGs, reproducing observed stable bimodal methylation patterns. Known recruitment of methylating enzymes by methylated CpGs could provide the necessary collaboration, but we predict that recruitment of demethylating enzymes by unmethylated CpGs strengthens inheritance and allows CpG islands to remain hypomethylated within a sea of hypermethylation.

## INTRODUCTION

Enzymatic modification of cytosine residues in eukaryotic DNA by the addition of a methyl group at the C5 position is thought to provide a stable and heritable chromatin mark that can be used to program alternative gene expression states (1–3). In vertebrates, 5 mC occurs primarily at 5′-CG-3′ sequences or CpG dyads. CpGs occur at low density across most of the mammalian genome, except for patches of ∼500–2000 bp termed CpG islands (CGIs) that are often associated with promoter regions (3). In adult mammals, CpGs outside CGIs are predominantly methylated, whereas CpGs within islands tend to be largely unmethylated. However, some CGIs appear to have two alternative stable methylation states—with the CpGs predominantly unmethylated in some cell types and predominantly methylated in other cell types, with methylation of promoter-associated CGIs correlating with promoter inactivity (4,5). The maintenance of the two alleles of a CGI within the same cell in distinct methylation states through cell division, as seen in X chromosome inactivation and genomic imprinting (6–8), supports the idea that these alternative methylation states represent a heritable chromatin mark. It is proposed that during development, transient signals cause methylation or demethylation of specific CGIs and this methylation status is then maintained and inherited through DNA replication even in the absence of the original signals. Failure to properly maintain and transmit DNA methylation states is associated with aberrant gene expression and disease (9–11).

For many years, the inheritance of these DNA methylation states has been rationalized by an elegant model (which we call the *standard model*) for the inheritance of the methylation state of a *single* CpG site (12,13). Because unmethylated cytosine is inserted into the new strands during DNA replication, a fully unmethylated CpG dyad naturally produces unmethylated CpGs in the daughter DNAs. However, replication of a fully methylated CpG site generates two hemimethylated sites. In the standard model, these new hemimethylated sites are recognized with high efficiency by maintenance methylases and rapidly restored to full methylation, thus maintaining the methylated state of the CpG for both daughter DNAs. Mammalian DNA methyltransferase 1 (DNMT1) seemed to be a good candidate for a maintenance methylase, having up to a 100-fold preference for hemimethylated versus unmethylated CpGs *in vitro* (14). The other recognized methylases in mammals, DNMT3A and DNMT3B, act similarly on unmethylated and hemimethylated CpGs *in vitro* (15,16). Thus, the activity of these ‘*de novo*’ methylases has been assumed to be under tight control *in vivo*, so that they can be used to establish methylation patterns at specific times and places without interfering with general inheritance of unmethylated CpGs.

However, there is now substantial evidence that these enzymes do not have the efficiency and specificity required for the standard model to work. The existence of substantial densities (at least 5%) of hemimethylated CpGs within methylated CGIs, revealed most precisely by hairpin-bisulfite PCR (6,7,17), shows that maintenance methylation is far from 100% efficient. Also, unmethylated DNA that is introduced into cells can become methylated even in the absence of DNMT3A and DNMT3B, showing that DNMT1 (or some other enzyme) has significant de novo activity *in vivo* (18). DNMT3A and DNMT3B are continuously needed for maintenance (1,19), raising the question of how they are prevented from acting generally on unmethylated CpGs. In addition, methylated CpGs are exposed to active enzymatic demethylation pathways (20). Thus, DNA methylation is more dynamic than envisioned in the standard model.

Using systematic scanning of methylation and demethylation reaction parameters and stochastic simulations of clusters of CpGs, we confirm previous theoretical modeling (21–23), showing that in the absence of perfect efficiency and specificity, the standard model cannot maintain distinct methylation states. In contrast, we show that true bistability can be achieved in dynamic systems where CpG sites collaborate—i.e. where the methylation reactions at one CpG are affected by the methylation status of nearby CpGs. Positive feedback collaborative reactions, in which methylated CpGs recruit methylases and unmethylated CpGs recruit demethylases, can produce strong heritable bistability. We show also that a collaborative model can generate systems that are sensitive to CpG density, so that bistable high-density CpG islands can exist in a sea of low-density methylated CpGs. Our results argue for a paradigm shift to underpin a new examination of the maintenance and inheritance of DNA methylation states.

## MATERIALS AND METHODS

**Figure 1.**
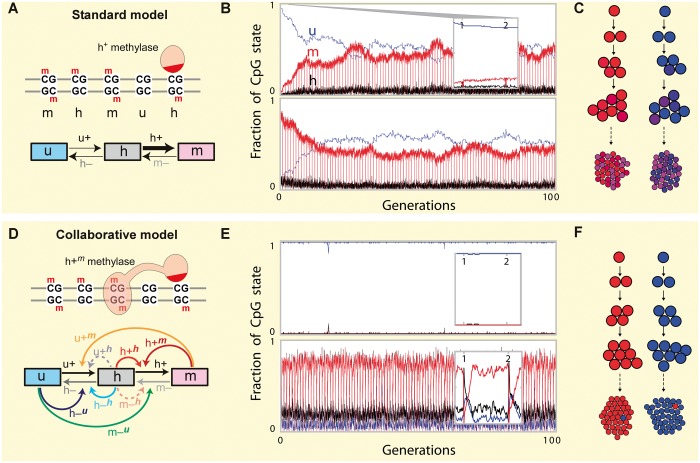
Standard and collaborative models for DNA methylation. (**A**) The standard model includes only simple transitions between fully methylated (*m*), hemimethylated (*h*) and unmethylated (*u*) CpG sites. (**B**) Time course for a typical standard model reaction scheme, tracking the changing fractions of *m* (red), *h* (black) and *u* (blue) CpG sites within the 80 CpG island >100 generations. The island was initialized either in the *U* state (upper panel) or the *M* state (lower panel) but converges to an intermediate state *u≈m*≈1/2 in both cases after ∼20 generations. Replication at the beginning of each generation sets the density of *m* to 0, which is rapidly restored to nearly pre-replication levels by maintenance methylation, as shown in the inset expanding the period from generations 1–2. (**C**) In the standard model, CGIs initially in the *M* state (red) or in the *U* state (blue) become partially methylated (intermediate shades) in their descendant cell populations. (**D**) In the collaborative model, methylation and demethylation reactions are affected by the status of nearby CpG sites (curved arrows), e.g. the dark red *h+^m^* arrow specifies an *h* to *m* transition facilitated by local *m* sites, which could be achieved by recruitment of a methylase enzyme by *m* sites. (**E**) As (B) but for a typical reactions scheme with the collaborative model, showing that a hypomethylated state (*U* state) or a hypermethylated state (*M* state) can be stably maintained over many generations. The insets expand the period from generations 1–2, showing the replication-induced disturbance in methylation. (**F**) The collaborative model can stably maintain bimodal (polarized) methylation states of CGIs in a cell population.

We simulate a CpG island consisting of 80 CpG sites, i.e. a 1D chain of 80 sites that can be in either of three states: unmethylated (*u*), hemimethylated (*h*) and methylated (*m*; Figure 1A). The CpG states are interconverted by specific methylation (+) and demethylation (*−*) reactions that are iterated stochastically over the CpG island.

For the standard model, there are four reactions (Figure 1A). For any particular reaction scheme, each of these reactions is assigned a specific rate. In the simulation, the CpGs in the island are subjected to repeated reaction attempts, each as follows: (i) we choose one of the four possible reactions according to their respective rates by a standard Gillespie algorithm. (ii) We choose a target CpG site at random and then test if the reaction chosen in (i) is allowed for the configuration of that site (e.g. a *u + *reaction cannot change an *h* target). If the reaction is possible, the target is changed, else no action is taken.

Each cell generation comprised an average of *N_t_* = 100 reaction attempts per CpG, yielding 100 × 80 reaction attempts for the entire CpG island per generation. After each simulated generation, DNA replication is mimicked by creating a daughter DNA in which each parental *u* site remains a *u*, each parental *m* site becomes an *h* site and each parental *h* site becomes *h* or *u* with 50% probability. This daughter strand is then exposed to the next cell generation of reaction attempts. Simulations were continued for at least 100 generations.

The collaborative model has an additional eight collaborative reactions (Figure 1D), each with a defined rate. For these reactions (randomly chosen as earlier in text), we also choose a random second CpG site as the mediator of the reaction. For the reaction to occur, the target CpG and the mediator CpG must match the chosen reaction.

## RESULTS

### Limitations of the standard model

Allowing for active demethylation reactions, the standard model comprises four reactions that interconvert the three different types of CpG site, the three states *u*, *h* or *m* (Figure 1A). The *h+* reaction is the ‘maintenance’ reaction, the addition (+) of a methyl group to a hemimethylated CpG (*h*); the *u+* reaction is *de novo* methylation (Figure 1A). A critical feature of the standard model is that the reactions that occur at one CpG are independent of the state of other CpGs. Previous mathematical examinations of this kind of model have shown that it does not give true bistability (21–23). With non-zero rates of the *u+* and *h−* reactions, and some failure in the *h+* reaction after replication, a highly methylated cluster of CpGs and a lowly methylated cluster of CpGs gradually converge to the same equilibrium densities of *u*, *h* and *m*.

We re-examined the standard model by stochastic simulations of an island of 80 CpG sites (∼1 kb), iterating the four possible methyl addition or removal reactions (Figure 1A). We generated >10^5^ different reaction schemes by choosing different sets of reactions rates, and each scheme was tested for its ability to maintain hypermethylated (*M*) and hypomethylated (*U*) states through many cell generations (‘Methods’ and Supplementary Information).

The minimum allowed reaction rate was 10*^−^*^4^, which for the *u+* reaction gives a 1% chance of *de novo* methylation per *u* site per generation. The maximum allowed reaction rate was 1*−u+*. As expected, we found that all combinations of reaction rates tested gave steady convergence of the *M* and *U* states of the cluster over time. Distinct *M* and *U* states could not be sustained for >100 generations and usually decayed within 10 generations. An example time series is shown in Figure 1B, for a system started in the *U* state (upper panel) or the *M* state (lower panel). Schemes that remained distinct for longest were those with *h+* closest to 1 and *u+* closest to 0.

Mean field analysis of the dynamics in this type of system (Supplementary Information, Section 1) shows that an upper bound for the expected lifetime of the methylation state is 1/(*N_t__._u+*). That is, if the number of reaction attempts per CpG per generation *N_t_* = 100 and the *de novo* methylation rate *u +* = 10*^−^*^4^, then a maximum lifetime of 100 generations is possible.

Thus, if a CGI is set into the *M* state in a founder cell, then as the population of descendant cells increases, the CGIs will steadily lose methylation and the variance of their methylation densities will increase (Figure 1C). Eventually, some CGIs descended from the original *M* state CGI will attain the same methylation densities as CGIs descended from a CGI initially in the *U* state, and the epigenetic distinction between the two populations will be blurred.

Even in the most successful standard model schemes, the distributions of CpG methylation are difficult to reconcile with three types of observations from sequencing of clones derived from bisulfite treated genomic DNAs:

1. Densities of *m*, *h* and *u*—hairpin-bisulfite PCR studies (6,7,17), have shown low densities (fractions) of *m* and *h* at low-methylated CpG clusters (density of *m* + *h* < 0.01 of total CpGs), and substantial densities of *h* at high-methylated clusters (*h* > 0.05). Even the most sustained standard model schemes (i.e. ∼100 generation stability) produced much lower densities of *h* in the *M* state (*h* < 0.01) and none was able to keep *m* + *h* below 0.01 for more than three generations (e.g. Figure 1B).
2. Bimodal methylation densities—*in vivo* methylation patterns of CpG clusters are generally bimodal; clusters tend to be either highly methylated or poorly methylated (4,5). Under the standard model, such bimodality can only exist transiently, and it seems unlikely that this would produce such consistent genome-wide bimodal patterns.
3. Dynamics of bimodality—in the standard model, bimodality of methylation densities should reduce with time (Figure 1C). However, analysis of clones from a 26-year-old and a 68-year-old human show that bimodality is maintained (5). Furthermore, Lorincz *et al.* (18) demonstrated that the bimodality of DNA methylation can actually increase over time. Clones derived from genomic insertion of DNA that had been partially methylated *in vitro*, were either fully unmethylated (*U* state) or highly methylated (*M* state). Such behavior, with two distinct stable states separated by an unstable intermediate state, is incompatible with the standard model and is the hallmark of a truly bistable system.

### A collaborative model

True bistability can be achieved in systems with positive feedback (24–27). In the case of DNA methylation, positive feedback would occur if the probability of a CpG becoming or remaining methylated is increased if other CpGs in the cluster are methylated; or conversely, if the probability of a CpG becoming or remaining unmethylated is increased by unmethylated CpGs in the cluster. We use the term collaborative for CpG reactions that are dependent on the methylation status of other CpGs. Collaboration between CpGs can readily be achieved by recruitment of the methylase or demethylase enzymes, i.e. if the enzymes bind directly or indirectly to CpGs in a particular methylation state and are able to catalyse reactions on nearby CpGs (Figure 1D). Collaboration by recruitment is also the basis of models for heritable bistability in nucleosome modification states (24). A number of observations are consistent with CpG recruitment of methylation enzymes (28–33). For example, DNMT1 is known to interact with the UHRF1 protein, which binds preferentially to hemimethylated DNA (29,30).

To test if collaboration between CpG sites can generate heritable bistability, we modified our parameter scanning simulations by adding eight collaborative reactions to the four non-collaborative reactions of the standard model (Figure 1D). We excluded the four possible collaboration reactions where *u* sites stimulate methylation and *m* sites stimulate demethylation, as such ‘self-destruction’ reactions should be strongly disfavored (26). For each reaction attempt, we again choose one of the 12 possible reactions according to their relative rates and a target site. However, if the chosen reaction is one of the collaborative links, we randomly select another CpG site to serve as a mediator of the reaction (enzyme recruiter). For example, if the *u+^m^* reaction is selected along with a target *u* site, then the *u→h* reaction proceeds only if a mediator *m* site is chosen. We again made 100 reaction attempts per CpG per generation. We kept *u+ ≥ *10*^−^*^4^ and *h+ ≥ *10*^−^*^4^; the bound on *u+* allowing explicit comparison with the analysis of the standard model.

Sampling more than 10^8^ combinations of reaction rates (Supplementary Information Section 2), we obtained many reaction schemes that gave at least 1000 generation stability for both the *M* and *U* states of the system. Such schemes resist drift of the *U* and *M* states, showing true bistability (Figure 1E). The *M* state system is perturbed by DNA replication but methylation is restored before the next DNA replication (Figure 1E, lower panel inset). Intermediate states are avoided, and the rare transitions between *U* and *M* states occur rapidly, within one single generation. A stability of 1000 generations means that a founder cell with a CGI in the *M* state could produce a population of 1000 descendants before the CGI flips to the *U* state in one of them. The descendants of this *U* cell would remain in the *U* state, so that descendant populations would eventually become mosaics for the *M* or *U* state of the CGI (Figure 1F). Thus, unlike the standard model, bimodality is a permanent characteristic of these bistable collaborative systems.

### Requirements for collaborative bistability

**Figure 2.**
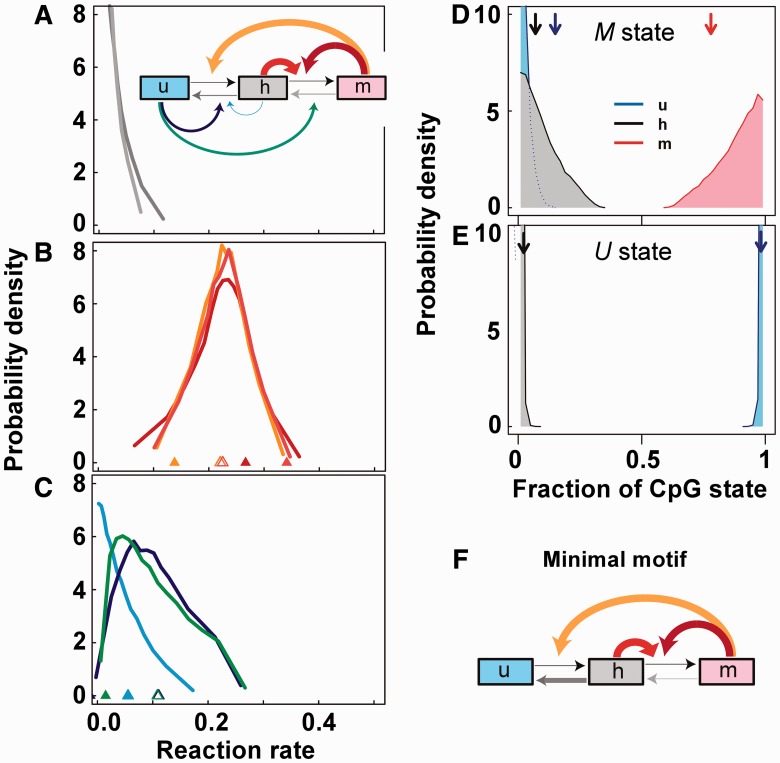
Features of bistable collaborative schemes. (**A–C**) Relative probability distributions of reaction rates among >10^8^ sampled collaborative reaction schemes that achieved 1000 generation stability of *U* and *M* states (Supplementary Information Section 2). The likelihood of obtaining a bistable solution was 0.015%, giving over 15 000 bistable schemes. (A) Low rates for the non-collaborative rates *h−* and *m−* are favored. (B) Collaborative methylation rates *h+^h^*, *u+^m^* and *h+^m^* near 0.2 are favored. (C) Collaborative demethylation rates *h−^h^*, *h−^u^*, *m−^u^* near 0.05 are favoured. The reaction diagram in A indicates the mean strengths of the corresponding reactions by the arrow thickness. Triangles in B and C indicate the mean of each reaction rate in all schemes (open) or in schemes with *u*/*h*/*m* densities matching experimentally observed densities: *u* = 0.15, *h* = 0.07 and *m* = 0.78 (6). (**D** and **E**) Relative probability distributions of average steady state fractions of *u*, *h* and *m* sites for each scheme in the *M* state (D) or the *U* state (E), showing that these schemes can produce experimentally observed fractions (vertical arrows). (**F**) The minimal motif able to achieve bistability combines the three favorable collaborative methylation reactions.

Analysis of the collaborative reaction schemes that were able to produce 1000 generation stability revealed characteristic strengths for each of the reactions. Figure 2A–C shows the probabilities of these schemes having particular reaction strengths. Low rates of the non-collaborative reactions (*u+*, *h+*, *m−*, *h−*) reactions are favoured (<10^−^^2^ for *u+* and *h+* and <0.1 for *m−* and *h−*; Figure 2A). That is, reaction schemes that have low rates for these reactions are more likely to be bistable (Figure 2A). These reactions are noise-like, producing CpG transitions independently of the overall *U* or *M* state of the cluster, and reduce the positive feedback in the system. Notably, the collaborative systems can tolerate much higher rates of the *u+*, *m−* and *h−* reactions than the standard model. However, although in the standard model the *h+* maintenance reaction needs to be strong, in the collaborative systems this reaction is weak so that the maintenance function is instead carried out by the positive feedback *h+^h^* and *h+^m^* reactions.

The rates for the three collaborative methylation reactions (*h+^h^*, *h+^m^* and *u+^m^*) were all optimal near 0.2 (corresponding to at least 20 reaction attempts per CpG site per generation; Figure 2B). These reactions should be strong enough to maintain the *M* state, but not so strong that they destabilize the *U* state.

Two collaborative demethylation reactions, *h−^u^* and *m−^u^* also have optimal rates; however, at values ∼0.05, substantially lower then the collaborative methylations (Figure 2C). These reactions provide positive feedback for the *U* state and need to be strong enough to maintain that state but not so strong that they destabilize the *M* state. The imbalance in the rates of the *h−^u^* and *m−^u^* reactions versus the *h+^h^*, *h+^m^* and *u+^m^* reactions counteracts the asymmetry in the system due to DNA replication, which provides a strong force in the *U* direction.

Some collaborative reactions corrupt bistability. The DNA replication asymmetry forces *m* and *h* sites to act as a team against *u* sites. Thus, the *h−^h^* reaction is disfavored (Figure 2C) because it is essentially a self-destruction reaction for the *m/h* team, acting to impede recovery of the *M* state after DNA replication. The *u+^h^* collaborative methylation reaction should also be weak; in the presence of some *u+* noise, this reaction is a powerful antagonist of the *U* state.

Figure 2D and E shows the probabilities of finding specific steady-state fractions of *u*, *h* and *m* in the *M* and *U* states of the bistable systems. The bistable collaborative schemes were much better than the standard model at reproducing the substantial densities of *h* and *u* sites observed in the *M* state, and the low densities of *h* and *m* sites seen in the *U* state (6,7,17).

To determine which reactions are critical for bistability, we also investigated motifs in which one of the reaction types was removed by fixing the corresponding reaction rate to zero (Supplementary Information Section 2), and again performing parameter scanning to find reaction schemes able to give 1000 generation bistability. We found that the *h+^h^*, *h+^m^* and *u+^m^* collaborative methylation reactions are all essential for bistability, whereas all other collaborative reactions could be removed. A ‘minimal’ motif with just these three collaborative reactions, combined with non-collaborative demethylation (Figure 2F) was also able to give 1000 generation bistabilities (Supplementary Information Section 3 and Supplementary Figure S5).

One potential disadvantage of a bistable system for inheritance of DNA methylation is the associated metabolic cost, as these systems are dynamic, involving back-and-forth reactions. In our modeling, we allow 100 reaction attempts per CpG per generation. If bistability required each CpG to undergo 100 methylation or demethylation reactions per generation, then the cost of bistability might be prohibitive. However, we found that the collaborative schemes do not require large numbers of ‘completed’ reactions to achieve bistability, with on average 3.3 and 0.03 reactions per CpG per generation needed to maintain the *M* and *U* states, respectively (Supplementary Information; Supplementary Figure S3).

We also examined the applicability of the model to larger CpG islands and found that in general, larger CpG islands allow for higher stability of *M* and *U* states because of a reduced contribution of stochastic fluctuations (Supplementary Information).

### Quantitating the robustness of collaborative bistability

**Figure 3.**
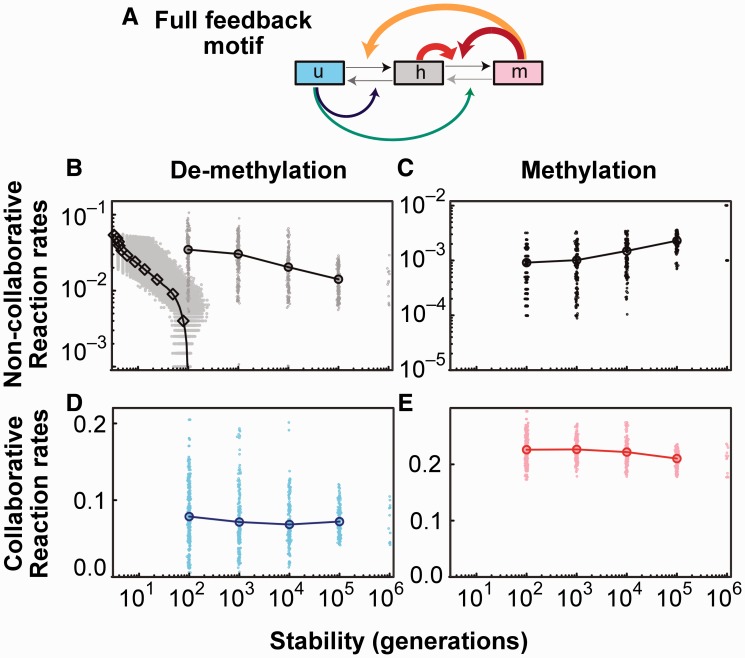
Robustness of collaborative bistability. (**A**) Full-feedback motif combining the five favorable collaboration reactions. (**B–E**) Reaction rates among schemes capable of increasing levels of bistability (stable maintenance of *U* and *M* states for indicated number of generations). The non-collaborative methylation reaction rates *u+* and *h+* were set ≥ 10*^−^*^4^ in the collaborative model. In the standard model, *u+* was set = 10^−4^ (Supplementary Information Section 2) (B) Gray points show mean non-collaborative demethylation reaction rates (*h− + m−*)/2 for individual schemes. The average over all schemes is shown by circles (collaborative schemes) or diamonds (standard model). (C) Average non-collaborative methylation reaction rates (average of *h+* and *u+*). These were fixed to either of two values for the 10^6^ generation testing to reduce computation. (D) Average collaborative methylation reaction rates (average of *h+^h^*, *u+^m^* and *h+^m^*). (E) Average collaborative demethylation reaction rates (average of *h−^h^*, *h−^u^*, *m−^u^*).

Combining the three essential collaborative methylation reactions with the two favorable collaborative demethylation reactions (Figure 2C) gives a motif where there is positive feedback collaboration in both the methylation and demethylation directions (Figure 3A). This full-feedback motif bears strong similarities to the optimal motif in our model for heritable bistability in nucleosome modification states (24), except for the presence of the *h+^h^* reaction. This reaction (and the higher strength of the collaborative methylation reactions) compensates for the *U*-favouring effect of DNA replication, whereas DNA replication did not favour one or other state in the nucleosome model.

We were curious whether this full-feedback motif could generate *U* and *M* state stabilities beyond 1000 generations. To test this, we used an iterative parameter sampling procedure in which successful reaction schemes were subjected to random small alterations in reaction rates and increasingly stable schemes were selected (Supplementary Information Section 2). By this refinement approach, we were able to find combinations of reaction rates capable of 10^6^ generation stability. The reaction rates for the collaborative reactions (Figure 3D and E) remained clustered near the optimal values seen in Figure 2B and C. Surprisingly, these optimized reaction schemes were able to tolerate substantial levels of the non-collaborative ‘noise’ reactions (Figure 3B and C). Although we have not tested for higher stabilities, it appears that substantially higher bistability could be obtained by further refinement. In contrast, the standard model with fixed optimal non-collaborative methylation rates *u+* = 10*^−^*^4^ is intolerant to demethylation noise (Figure 3B).

We also examined the minimal motif (Figure 2F) for extended bistability (Supplementary Figure S4). Although 10^4^ generation stability was possible, it was a factor of 100 less likely than the full-feedback motif (Figure 3A), pointing to a strong, stabilizing effect of collaborative demethylation.

### Bistable islands in a sea of methylation

So far, we have only considered an isolated CpG island without interaction with its surroundings. We explored whether certain collaborative systems were able to reproduce a larger scale methylation pattern, i.e. where CpG islands can exist in stable *M* or *U* states while the surroundings remain stably methylated.

In the modeling up to this point, we have used what we term global interactions—the target and the mediator can be any CpGs in the cluster with equal probability. However, when simulating a CpG island and an adjacent non-island, we found that applying such unrestricted spatial interactions prevented stable maintenance of differences between the regions. In a previous study of nucleosome modification, we showed that restricting interactions to nearest-neighbor sites (local collaboration) and adding a distance constraint to global collaboration could be used to help demarcate nucleosome modification patterns and still retain bistability (34). We found that introducing such spatial restrictions and using the difference in CpG density between islands and outside regions (35) allowed distinct behavior for the island and a neighboring region.

**Figure 4.**
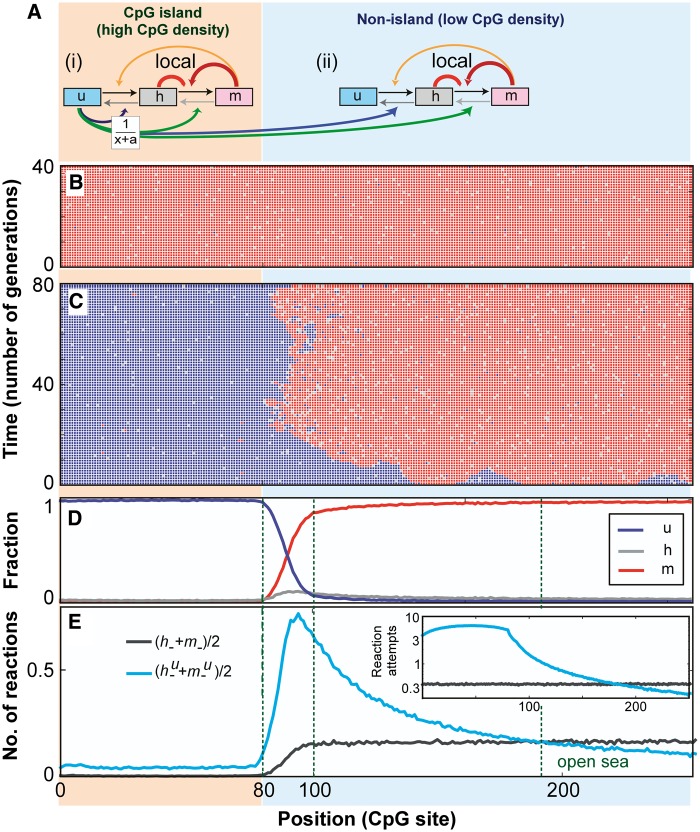
CpG island adjacent to a low-density CpG region. (**A**) Schematic of a spatially restricted collaborative motifs, showing reactions for the region (i) within the CpG island and (ii) beyond the CpG island. The collaborative methylation reactions act locally (nearest-neighbor interaction), whereas demethylation reactions are only mediated by unmethylated CpG sites within the CpG island. Demethylation reactions act globally, but collaboration probability decays with distance (see text). Arrow thickness represents approximate relative reaction rates for reaction schemes that were able to generate 1000 generation bistability for the CpG island, although the adjacent low CpG density ‘sea’ remained hypermethylated. (**B** and **C**) Space-time plots (vertical time axis) for a typical bistable system, showing *m* (red), *h* (gray) and *u* (blue) sites when the CpG island is in the *M* state (B) or the *U* state (C). The system comprised 80 island CpG sites and 240 adjacent non-island CpGs (only 160 shown). (**D**) Time averages (>4000 generations) of *u*, *h* and *m* densities at each CpG position. (**E**) Average number of reaction completions per CpG per generation (>4000 generations) for non-collaborative reactions, (*h− + m−*)/2, and collaborative demethylation reactions, (*h−^u^ + m−^u^*)/2, as a function of position. Vertical lines distinguish various regions near the CpG island. Inset to E: number of reaction attempts per CpG per generation.

First, the collaborative methylation reactions were made local (Figure 4A), meaning that collaborating CpGs must be nearest neighbors on the DNA. That is, a methylase enzyme recruited by a mediator *m* site, for example, can only act on the next CpG site along the DNA, on one side or the other.

Second, we added a DNA distance constraint on the collaborative demethylation reactions, making the interaction probability decrease with increasing DNA separation. Such decay of contact probability is seen *in vitro* and *in vivo* and can be approximated by a linear decay 1/*x*, where contact probability reduces as the inverse of DNA separation *x* (36,37). A DNA distance unit of 1 is used between CpG sites within the island and 10 between CpG sites outside the island, to reflect the relative densities of CpG sites (35). To mimic the stiffness of DNA over short distances, we included a ‘minimal’ distance factor, *α* = 20 (roughly a one nucleosome spacing), such that contact probability decays as 1/(*x* + *α*).

Third, the low CpG density outside islands is likely to strongly disfavor the collaborative demethylation reactions in these regions, and we assumed that the collaborative demethylation enzymes are only recruited effectively to high-density *u* sites and are thus restricted to CpG islands. Such CpG site density-dependent recruitment can be rationalized if recruitment is a locally cooperative reaction, requiring nearby CpG sites for binding. Some support for this assumption comes from the observation that the TET1 protein, which appears to catalyze a critical demethylation reaction, tends to be localized to high-density low-methylation CpG regions (38–40).

With these spatial restrictions, we simulated a high-density 80 CpG island adjacent to a low-density region of 240 CpG sites. We again performed parameter sampling (Supplementary Information Section 2) to find combinations of reaction rates that allow 1000 generation bistability of the island (stable *U* or *M*) and a persistent *M*-state in the adjacent ‘sea’. Figure 4B and C show the evolution over time of a typical solution. These space-time diagrams (note that time is the vertical axis) show that the CpG island can exist in a stable *M*-state that is not distinguishable from that of the CpG open-sea (Figure 4B). With the same set of reaction rates, the CpG island can also stably remain in the *U*-state, while approximately 20 sites away from the edge of the CpG island, the *M*-state is again present (Figure 4C). Hence, we find that collaborative reactions, combined with a dependence of these reactions on CpG site density can allow for bistability within the CpG island but overall dominance of hypermethylation outside the island.

Fluctuating densities of *m* and *u* sites (Figure 4C) form an extended transitional regime between the island in the *U* state and the *M* sea, reminiscent of CpG shores (41) (Figure 4D; DNA distance is 10× longer outside the island, as the CpGs are further apart). These fluctuations are driven by competition between methylation and demethylation positive feedback that are balanced in this region. This competition produces a zone of pronounced hemimethylation at the boundary of the CpG island that should be experimentally testable by hairpin bisulfite PCR. The fluctuations can be persistent for many generations (Figure 4C), potentially generating long-lived aberrant gene expression states (41).

Although there is no strong evidence for the particular spatial constraints that we have imposed on the collaborative reactions, our analysis shows, as for nucleosome modification systems (34), that collaborative systems can in principle provide spatial patterning and retain heritable bistability without invoking specific boundary elements.

## DISCUSSION

Our modeling shows that collaboration between CpG sites can provide strong stable inheritance of alternative DNA methylation states of a cluster of CpGs. Collaborative bistability is more consistent with existing experimental data than the widely accepted standard model for methylation maintenance. (i) It does not require an unlikely degree of specificity and efficiency of the methylation enzymes. (ii) It naturally produces the observed bimodal pattern of DNA methylation levels. (iii) It naturally produces the bistable behavior seen in certain experiments. (iv) It can produce the observed densities of unmethylated, hemimethylated and fully methylated CpGs seen in both low- and high-methylation states. (v) It is strengthened, rather than weakened, by the existence of demethylation reactions. (vi) It can, with additional constraints, reproduce spatial features of CpG methylation in the chromosome.

Our modeling also makes a number of predictions about the nature of CpG collaboration, some of which are supported by existing data. In particular, our analysis predicts that three collaborative methylation reactions are critical. Methylated CpGs should stimulate methylation of nearby unmethylated and hemimethylated sites (*u+^m^* and *h+^m^* reactions) and hemimethylated CpGs should stimulate the full methylation of other hemimethylated sites (*h+^h^* reaction). *In vitro* experiments with DNMT1 provide evidence for the *u+^m^* reaction, and presumably the *h+^m^* reaction; domains of DNMT1 that are distinct from its catalytic domain bind to *m*-containing DNA (28), and DNMT1 methylation of *u* sites *in vitro* is stimulated by the presence of *m* sites in cis (33). The *h+^h^* reaction appears to be provided by the interaction of DNMT1 with the hemimethylated CpG binding protein UHRF1 (29,30). The *de novo* methylases DNMT3A and DNMT3B are preferentially bound to nucleosomes at regions of high-methylation density, potentially providing additional *u+^m^* activity (32).

Importantly, our modeling predicts that the non-collaborative *h+* methylation reactions should be rare. This seems to be the case, although DNMT1 activity *in vivo* appears to be highly dependent on UHRF1 (29,30). The collaborative *u+^h^* reaction should also be weak to protect low-methylation CpG clusters, where *h* sites are occasionally produced. Thus, the *h* versus *u* preference of DNMT1 means that its recruitment by UHRF1 should not provide this reaction. However, the *u+^h^* reaction would be provided through the reported interaction of the *de novo* methylases DNMT3A and DNMT3B with UHRF1 (31). Accordingly, we expect that there is either some mechanism to prevent *de novo* activity of these enzymes when recruited by UHRF1 or that their recruitment (or UHRF1 binding) requires multiple *h* sites, such as would only be found in high-methylation CpG clusters.

Although we have not modeled it explicitly, collaboration could also work in more indirect ways, for example, by reciprocal feedback between specific nucleosome modifications and DNA methylation enzymes (1,42), as proposed for maintainance of CHG methylation in plants by recruitment of the CMT3 DNA methylase by the H3K9me2 mark coupled with recruitment of the SUVH4 H3K9methylase by methylated CHG (43). The stability and inheritance of cytosine methylation in non-CpG contexts (44,45) has been less studied than CpG methylation and may involve its own collaborative processes or may interact with CpG collaboration.

Although we found that two collaborative demethylation reactions (*m−^u^* and *h−^u^*) are not critical for bistability, they improved stability and provided a means of achieving the spatial patterning of DNA methylation. We thus predict that unmethylated CpGs will stimulate demethylation of nearby methylated and hemimethylated CpGs. The TET-family proteins are possible mediators of such collaboration. These proteins oxidize 5-methyl cytosine to 5-hydroxymethyl cytosine and other products that are substrates for repair pathways, leading to base removal and replacement with unmethylated C (46–48). Tet1 associates with CpG islands *in vivo* (38–40) and Tet1 and Tet3 contain CXXC motifs, which in other proteins confer specific binding to unmethylated CpGs. However, the isolated TET CXXC domains do not share this specificity (40,49,50) Demethylation may also be initiated by deamination of 5mC by APOBEC family proteins, such as AID (51), with unmethylated cytosine again inserted by repair pathways (20). Whether APOBEC proteins are targeted in a CpG methylation-specific manner is not known. Thus, though there is evidence for active demethylation pathways, it is not yet clear whether they involve CpG collaboration.

Collaboration could be tested *in vivo* by using a genomic insertion system [e.g. (18,52)] to insert synthetic sequences of differing CpG densities and varying levels of premethylation, with analysis of resulting clones by hairpin-bisulfite PCR sequencing to determine methylation status, as well as by ChIP techniques to determine recruited proteins. Collaborative effects of CpGs could be further tested *in vitro* using specifically methylated ‘recruitment’ DNA fragments and methylated or unmethylated ‘target’ DNA fragments.

The requirements of a truly bistable CpG inheritance system are shared with those we have identified for epigenetic systems based on nucleosome modifications (24,34)—at least one of the positive feedback reactions in the system must be cooperative and dependent on sites beyond nearest neighbors. In the collaborative CpG model, cooperativity is provided indirectly by the 2-step modification processes (24) where the conversion of a *u* site to an *m* site requires the successive action of two *u* mediator sites. This requirement is likely to be fulfilled by the *u→h* and *h→m* reactions being carried out by different enzymes. Long-range interaction in CpG interactions is a reasonable assumption, given the prevalence of DNA looping interactions in other DNA-based processes (36,37), however has not been directly demonstrated in CpG methylation (or nucleosome modification).

Thus, we argue that it is time to fundamentally change the way we think about the maintenance and inheritance of DNA methylation—the focus should be on understanding how the methylation status of CpGs can affect the methylation and demethylation reactions at other CpGs.

## SUPPLEMENTARY DATA

Supplementary Data are available at NAR Online, including [6,35,53].

## FUNDING

Danish National Research Foundation (to J.O.H., C.L. and K.S.) through the Center for Models of Life. The project was partially supported by the Australian NHMRC [1025549]. Funding for open access charge: Danish National Research Foundation through the Center for Models of Life.

*Conflict of interest statement*. None declared.

## Supplementary Material

Supplementary Data
