# Supplementary material for: Collaboration between CpG sites is needed for stable somatic inheritance of DNA methylation states
Source: Nucleic Acids Res. 2013 Nov 27;42(4):2235–44. doi: 10.1093/nar/gkt1235 (PMC3936770; doi:10.1093/nar/gkt1235)
Supplement: Supplementary Data [file supp_42_4_2235__index.html]

Collaboration between CpG sites is needed for stable somatic inheritance of DNA methylation states — Collaboration between CpG sites is needed for stable somatic inheritance of DNA methylation states — Supplementary Data 

# Collaboration between CpG sites is needed for stable somatic inheritance of DNA methylation states

## Supplementary Data

files

**Files in this Data Supplement:**

- Supplementary Data - pdf file
