# Supplementary material for: Collaboration between CpG sites is needed for stable somatic inheritance of DNA methylation states
Source: Nucleic Acids Res. 2013 Nov 27;42(4):2235–44. doi: 10.1093/nar/gkt1235 (PMC3936770; doi:10.1093/nar/gkt1235)
Supplement: Supplementary Data [file supp_gkt1235_nar-02577-n-2013-File006.pdf]

Collaboration between CpG sites is needed for stable inheritance of DNA methylation states.
— SUPPLEMENTARY INFORMATION —

Jan O. Haerter[1], Cecilia Lövkvist[1], Ian B. Dodd[2], Kim Sneppen[1,*]

1 Center for Models of Life, Niels Bohr Institute, University of Copenhagen, Blegdamsvej 17, DK-2100, Copenhagen, Denmark

2 Department of Molecular and Biomedical Sciences (Biochemistry), University of Adelaide SA 5005, Australia

*** Correspondence: sneppen@nbi.dk**

Abstract

Here we provide additional analysis supporting the conclusions of the main article but not essential for its understanding. The general purpose of this supplementary information is to explore the standard model analytically (Section 1), to provide details of our parameter sampling (Section 2), and to offer several additional details on the robustness of our results (Section 3).

1 The standard model: mean field solution

The simple model as shown in Fig. 1A consists of four non-collaborative reactions (u_+, h_-, h_+, and m_-) and replication. Replication changes the state of all m-sites to h and the state of h-sites is converted at probability 1/2 to u. Replication was defined to occur only every N_t timesteps. We first discuss an approximation where replication is considered as a time-continuous process. This allows us to yield continuous equations for $u(t)$ and $m(t)$. The solution of this system allows us to define a hierarchy between the parameters, yielding the result that the parameter h_+ should be largest and u_+ minimal. Given this hierarchy, we then discuss an upper bound for the stability of the polarized methylation states.

We make the approximation of replication occuring continuously at every timestep, albeit rescaled by a factor $1/N_t$. When observing the timeseries in Fig. 1B this approximation seems quite good as the system after replication nearly returns to the densities present before replication, i.e. replication acts as a relatively small perturbation. In the mean field approximation – i.e. corresponding to an infinite number

of CpG sites – the time-dependence of $u(t)$ and $m(t)$ is given by

$$\frac{du(t)}{dt} = -u_+ u + h_- h + \frac{1}{2N_t} h \tag{1}$$

$$\frac{dm(t)}{dt} = h_+ h - m_- m - \frac{1}{N_t} m \ . \tag{2}$$

Using $h = 1 - m - u$, we have

$$\frac{du(t)}{dt} = -(u_+ + \tilde{h}_-)u - \tilde{h}_- m + \tilde{h}_- \tag{3}$$

$$\frac{dm(t)}{dt} = -(h_+ + \tilde{m}_-)m - h_+ u + h_+ \ , \tag{4}$$

where we have defined $\tilde{h}_- \equiv h_- + 1/2N_t$ and $\tilde{m}_- \equiv m_- + 1/N_t$. The latter replacement simply means that replication acts fto boost the effect of the demethylation reactions h_- and m_-. The above equations correspond to the system of ordinary linear differential equations

$$\frac{d\mathbf{x}}{dt} = \mathbf{A} \cdot \mathbf{x} + \mathbf{w} \ , \tag{5}$$

with the constant 2x2-matrix

$$\mathbf{A} \equiv \begin{pmatrix} -(u_+ + \tilde{h}_-) & -\tilde{h}_- \\ -h_+ & -(h_+ + \tilde{m}_-) \end{pmatrix} \tag{6}$$

and

$$\mathbf{w} \equiv \begin{pmatrix} \tilde{h}_- \\ h_+ \end{pmatrix} \ . \tag{7}$$

The solution to Eq. 5 is given by

$$\mathbf{x}(t) = c_1 \mathbf{v}_1 \exp \lambda_1 t + c_2 \mathbf{v}_2 \exp \lambda_2 t + \mathbf{b} \tag{8}$$

where

$$\lambda_{1/2} \equiv \frac{1}{2}\left(-r \mp \left(r^2 - 4(\tilde{h}_-\tilde{m}_- + u_+(h_+ + \tilde{m}_-))\right)^{1/2}\right) = \frac{r}{2}\left(-1 \mp \left(1 - 4q/r^2\right)^{1/2}\right) , \qquad (9)$$

are the eigenvalues of $\mathbf{A}$, $r \equiv u_+ + h_+ + \tilde{h}_- + \tilde{m}_- = 1 + 3/(2N_t) \approx 1$ is the sum of reaction rates and $q \equiv \tilde{h}_-\tilde{m}_- + u_+(h_+ + \tilde{m}_-))$,

$$\mathbf{v}_1 \equiv \left(\frac{u_+ - h_+ + \tilde{h}_- - \tilde{m}_- + r\left(1 - 4q/r^2\right)^{1/2}}{2h_+}, 1\right)^T , \qquad (10)$$

$$\mathbf{v}_2 \equiv \left(\frac{u_+ - h_+ + \tilde{h}_- - \tilde{m}_- - r\left(1 - 4q/r^2\right)^{1/2}}{2h_+}, 1\right)^T \qquad (11)$$

are the corresponding eigenvectors and

$$\mathbf{b} \equiv \frac{1}{q}\left(\tilde{h}_-\tilde{m}_-, u_+h_+\right)^T \qquad (12)$$

results from the particular solution of Eq. 5. $\mathbf{b}$ contains the steady state solutions of Eq. 5: Larger methylation rates u_+ and h_+ lead to larger steady state densities of the m-state while larger demethylation through $\tilde{h}_-$ and $\tilde{m}_-$ lead to larger levels of unmethylated sites.

The steady state density of the hemimethylated state is then just

$$h = \frac{u_+\tilde{m}_-}{q} , \qquad (13)$$

i.e. the product of the two rates adding to the h-state has to be maximized for large values of h.

The constants $c_{1/2}$ result from solving Eq. 8 for the respective initial condition, e.g. $u(0) = 1$ and $m(0) = 0$ in Fig. 1B, first panel.

The eigenvalues in Eq. 9 constitute the possible decay rates of a given methylation state and depend on the reaction rates in terms of q. We start by assuming q is maximal. One can yield a maximal $q \approx 1/4$ and this would make both eigenvalues equal as the square-root term in Eq. 9 will vanish. The eigenvalues then become

$$\lambda_{1/2} = -r/2 \geq 1/2 . \qquad (14)$$

This means, the system would collapse within only very few timesteps.

The other option is to minimize q. With $q \ll 1$ the term $(1-4q/r^2)^{1/2}$ in Eq. 9 can then be simplified to

$$(1-4q/r^2)^{1/2} \approx 1-2q/r^2+O((q/r^2)^2)\ . \tag{15}$$

The eigenvalues (Eq. 9) then become

$$\lambda_1 = -r + q/r \approx -r \approx -1 \tag{16}$$

and

$$\lambda_2 = -q/r\ . \tag{17}$$

Initial conditions

In Fig. 1B we show the time dependence for the two initial conditions, (i) $u(0)=1$, $m(0)=h(0)=0$ and (ii) $m(0)=1$, $h(0)=u(0)=0$, respectively. These initial conditions are used to determine the constants c_1 and c_2 in Eq. 8.

Using (i) in Eq. 8 we yield for the constants:

$$c_1 = \frac{u_+h_+(r-(r^2-4q)^{1/2})}{2q(r^2-4q)^{1/2}} \approx \frac{u_+h_+}{r^2}\frac{1}{1-2q/r^2} \ll 1\ , \tag{18}$$

$$c_2 = -\frac{u_+h_+(r+(r^2-4q)^{1/2})}{2q(r^2-4q)^{1/2}} \approx -\frac{u_+h_+}{q}\frac{1-q/r^2}{1-2q/r^2}\ , \tag{19}$$

where $|c_2| \gg |c_1|$ and the approximation again uses Eq. 15. Since c_2 dominates, $\mathbf{v}_2$ and $\lambda_2 = -q/r$ describe the dynamics of the methylation state and the decay of $u(t)$ is minimized when q becomes minimal. Similar considerations apply for the initial condition (ii) and again $c_2 \gg c_1$.

Discussion of decay rate

We have for the decay rate $\lambda_2 = -q/r$. Per generation, i.e. during N_t timesteps, this yields

$$N_t\lambda_2 = -N_t\frac{\tilde{h}_-\tilde{m}_- + u_+h_+ + u_+\tilde{m}_-}{1+3/(2N_t)}\ . \tag{20}$$

More explicitly, we have

$$N_t\lambda_2 = -\frac{N_t}{N_t+3/2}\left((h_-m_- + u_+h_+ + u_+m_-)N_t + \left(\frac{m_-}{2} + h_- + u_+ + \frac{1}{2N_t}\right)\right) . \tag{21}$$

In Eq. 21, the term $(m_-/2 + h_- + u_+ + 1/(2N_t))$ demands minimization of m_-, h_- and u_+ (compare also Fig. S1). Under the constraint of all reaction rates summing to unity, this can only be achieved by maximizing h_+, i.e. choosing $h_+ \approx 1$. In turn, large h_+ demands minimization of u_+ in u_+h_+ in Eq. 21. This consideration singles out u_+ as the critical noise parameter of the simple model and sets a scale for the perturbation in the collaborative model.

Limiting cases

(a) If no lower bound is specified for the reaction rates h_- and m_-, the resulting rates $\tilde{h}_-$ and $\tilde{m}_-$ are minimal and result directly from replication:

$$\tilde{h}_- = 1/(2N_t) \tag{22}$$

and

$$\tilde{m}_- = 1/N_t . \tag{23}$$

Inserting into Eq. 21 yields

$$N_t\lambda_2 = -\frac{N_t}{N_t+3/2}\left(u_+h_+N_t + u_+ + 1/(2N_t)\right) . \tag{24}$$

When $N_t \gg 3/2$ and taking $h_+ \approx 1$ we yield

$$N_t\lambda_2 \approx N_tu_+ + \frac{1}{2N_t} , \tag{25}$$

i.e. the typical decay time is proportional to the sum of the noise during one generation N_tu_+ and the inverse generation time. For fixed noise u_+, minimal decay would hence result for

$$N_t = \sqrt{1/2u_+} . \tag{26}$$

For a value of $u_+ \sim 10^{-4}$, optimal conditions would hence be met for $N_t \sim 10^2$. This means, as the noise level drops, one must adjust the number of timesteps per generation to achieve optimal lifetime. This can be seen as follows: Lower noise means, that less flux $u \to h$ occurs per generation, thus granting higher stability of a polarized u-state. However, the correspondingly longer lifetime must be matched by an equally faithful maintenance of the stable m-state between replication steps. This faithfulness can only be achieved by an increase of the number of timesteps to perpetually perform the reaction $h \to m$. We can then also compute the optimal lifetime of a polarized state. Substituting Eq. 26 in Eq. 25 we have for the optimal lifetime at a given noise levels

$$N_t \lambda_2 = \frac{3}{\sqrt{2}} \sqrt{u_+} \ . \tag{27}$$

Even under these optimal conditions, a noise level $u_+ \approx 10^{-7}$ would be required to yield 1000 generations of polarization.

(b) If the reaction rates h_- and m_- dominate over the effect of replication, i. e. replication occurs rarely compared to internal noise, $N_t \gg 1$, then it follows from Eq. 21

$$N_t \lambda_2 = -(h_- m_- + u_+ h_+ + u_+ m_-) N_t \approx -(u_+ + h_- m_-) N_t \ . \tag{28}$$

Hence, in this limit, minimizing the product of demethylation reaction rates would be optimal. Noting that for the simple model the most slowly decaying – i.e. optimal for persistant polarization of methylation – solutions require large values of $h_+ \approx 1 \gg \tilde{m}_-$, we can approximate q as

$$q \approx \tilde{h}_- \tilde{m}_- + u_+ h_+ \ . \tag{29}$$

With $u_+ \ll 1$, $h_+ \approx 1$ and the constraint of all reaction rates summing to unity, $q \ll 1$.

(c) We now return to the original system where replication does *not* occur continuously, but abruptly every N_t timesteps. To obtain a lower bound for the decay rate for that case, we assume $m_- = 0$ and $h_- = 0$. This yields that in the M-state, after replication strong h_+ allows all h sites to fully transition back to m, making $h = 0$. In this approximation, replication can simply be ignored and we need only

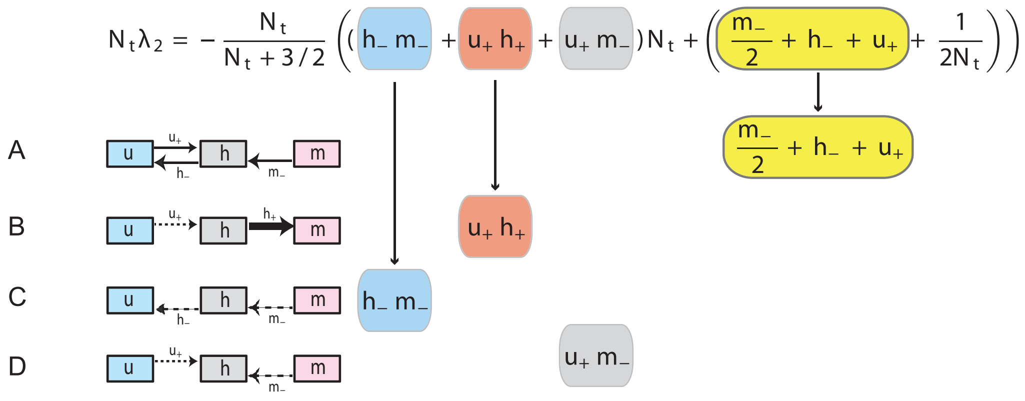

Figure S1: **Hierarchy of parameters.** (A) Minimizing the rates m_-, h_- and u_+ (compare schematic and terms in yellow bubble). (B) Result of A and normalizing constraint for the reaction rates demands large u_+. This requires particular minimization of u_+, the overall noise term. (C) Further, either h_- or m_- should be minimized to reduce term in red bubble. (D) Remaining term will be automatically small.

consider the decay of the U-state. The solution is then given by Eq. 28 with $h_- = m_- = 0$, hence

$$N_t \lambda_2 = -u_+ N_t \ . \tag{30}$$

Hence, the above theoretical analysis of the dynamics shows that in this type of system, maintenance of polarized M or U states can only be obtained by a large h_+ rate, $h_+ \approx 1$, as well as minimal rates for the detrimental noise rates u_+, m_- and h_-. Unlimited maintenance of the U state could only be yielded when $u_+ = 0$. In the extreme limit where $u_+ \ll 1$ and both h_- and m_- vanish, an upper bound for the expected lifetime of the methylation state is $1/N_t u_+$ i.e. a lifetime of 100 generations is possible for $N_t = 100$ and $u_+ = 10^{-4}$.

2 Parameter Sampling

To allow for unbiased model testing, we performed random sampling of the space spanned by the parameters corresponding to the reactions shown in Fig. 1D, main text. Within a CpG island the average

CpG frequency is 10 bp per CpG and the length of the island varies but is on average approximately 1 kb [Antequera, 2007, Takai and Jones, 2002]. We found that larger system sizes tend to become more stable in the collaborative model. To have a strict test of our theory, we therefore generally chose a somewhat smaller system size of 80 sites, but explore the effect of system size below.

In a pre-screening of parameters it was found that u_+ and h_+ are noise-like, i.e. their main effect is to de-stabilize the corresponding motif when their value is large. We therefore sample these from decaying exponential distributions. In particular, we choose them to take any of the discrete values $\{0.0001, 0.0002, 0.0004, 0.0008, 0.0016\}$ at equal probability, where the magnitude is expressed relative to the sum of all other parameters. The results confirm that all models prefer these values to be small, i.e. for working solutions 0.0001 is the dominant choice. To be able to compare the relative strength of all other parameters, we sampled these uniformly in the range $[0, 1]$ and then expressed them relative to the overall sum of rates. A relative shift of the resulting distributions of the different parameters — such as shown in Fig. 2 of the main text for the collaborative methylation reactions — then indicates that one rate, e.g. h_+^m in the figure, is preferably large compared to the others. The sampling of parameters is also summarized in Tab. S1.

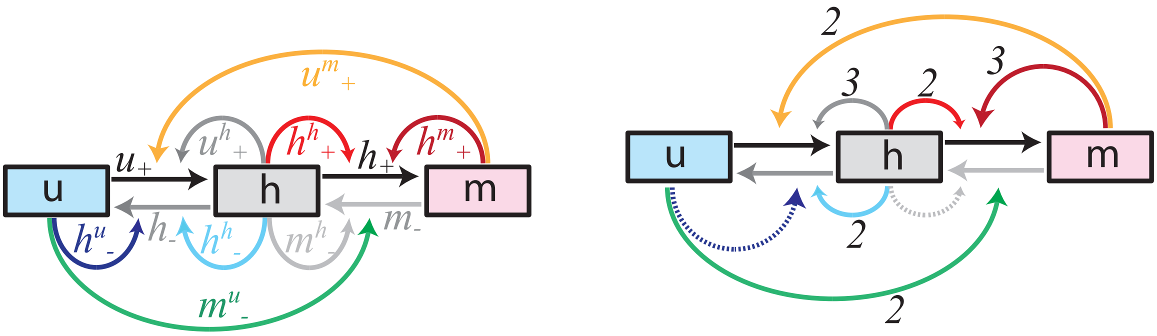

Figure S2: Number count of distinct motifs. Reproduction of the motif in Fig. 1D and number count of distinct reactions.

To reduce the numerical effort of the sampling, we have further considered several pairs of reactions as redundant. For example, the reaction pairs $\{h_-^u, u_+^h\}$ or $\{m_-^h, h_+^m\}$ are identical up to a change of sign. This becomes clear when considering that these reactions correspond to a mean field contribution $u \cdot h$ and $h \cdot m$, respectively.

When sampling, we therefore only consider the difference of the two parameters as the random variable

parameter name	parameter range	parameter sampling
u_+, h_+	0.001–0.016	exponential
all other reactions	$0-1$	uniform

Table S1: Parameters and their sampling

and allow this to vary in the range $[-1, 1]$. This type of simplification is acceptable as long as it is kept in mind that the noise – introduced by back-and-forth reactions of both reactions within the pair – is captured already by the noise terms u_+, h_+, etc.

To obtain the number count of distinct reactions, we return to Fig. 1D, main text, reproduced in Fig. S2 along with the number count of each reaction. A motif results from a given set of links being present. To obtain the total number of distinct motifs, we multiply the number of individual configurations each link can take on: The non-collaborative reactions (u_+, h_+, h_-, and m_-) are present in all motifs, i.e. they do not contribute in the multiplication. The reactions u_+^m, h_+^h, h_-^h, and m_-^u can either be set to finite, positive values, or be set to zero. They therefore each contribute a factor of 2 to the total number of configurations. The reaction u_+^h is the inverse of the reaction h_-^u (as stated above). Therefore, for this pair of reactions there are three possible configurations: (i) $h_-^u > u_+^h$, effectively corresponding to the presence of h_-^u. (ii) $h_-^u < u_+^h$, effectively corresponding to the presence of u_+^h. (iii) $h_-^u = u_+^h$, effectively corresponding to the absence of both h_-^u and u_+^h. A similar argument applies to the pair $\{m_-^h, h_+^m\}$, again yielding a factor of three. Multiplying all possible configurations, we obtain $2^4 \cdot 3^2 = 144$ distinct motifs. Note however, that we are of course sampling a much larger number of distinct parameter *combinations*, as parameters are sampled in high resolution along the ranges presented above. In total, we have sampled far more than 10^8 distinct parameter combinations. The simulation then proceedes as follows: (i) We fix a random sample of parameters as described above. (ii) We demand stability of the M state by performing the required number of N_t timesteps per generation. We simulate replication by performing the shift of $m \to h$ for all m sites and the shift $h \to u$ for each h site at probability 1/2. We repeat this procedure for all required generations. The density of m must be larger than either of the two other densities at the end of each performed generation, i.e. immediately before the subsequent replication step. (iii) We demand stability of the U state by a similar procedure. Additionally, we demand even stricter fulfillment of stability by requiring that the U state be sustained also without the replication step, which generally strengthens the U state.

We follow several precautions to guarantee robustness of the results:

(i) Replication can reinforce the stability of the U state, so to select against this type of marginal stability, we demanded stability of the U state for > 100 generations without replication.

(ii) To avoid slowly drifting U states, another seemingly stable state, the mean methylation densities after every generation were tested for significant differences.

Further, we comment on the number of reactions in the collaborative model: One potential disadvantage of a dynamic system for inheritance of DNA methylation is the associated metabolic cost of back-and-forth reactions. However, the average number of completed reactions per CpG per generation among collaborative schemes that achieved 1000-generation bistability is much less than the number of reaction attempts and not substantially more than for the standard model (Fig. S3). In this figure, a comparison of numbers of reactions per CpG site per generation in the U and M states for different models is shown. Both noisy models share $u_+ = 10^{-4}$ and $h_- = m_- = 0.005$. The standard noisy model uses $h_+ = 1 - h_- - m_- - u_+$; collaborative models sample all remaining parameters at random.

Reactions per CpG per generation

Methylation state	U	M
Error free models (no noise)	0	1
Standard model (noise)	0.26	1.7
Collaborative model (noise)	0.03	3.3

Figure S3: **Numbers of reactions.** Comparison of numbers of reactions per CpG site per generation in the U and M states for different models. Both noisy models share $u_+ = 10^{-4}$ and $h_- = m_- = 0.005$. The standard noisy model uses $h_+ = 1 - h_- - m_- - u_+$; collaborative models sample all remaining parameters at random.

Iterative Parameter Sampling (IPS)

For the parameter sampling in Fig. 3, main text, we made use of an iterative procedure, which we call *iterative parameter sampling*, or IPS. IPS can be described as follows: (i) For a given acceptance criterion,

in this case achieving 100 generations of bi-stability, select a number n_{sol} of working motifs as described above. Each of these solutions is characterized by a unique numerical parameter vector v_i. (ii) Increased strictness of acceptance and perturbation of parameters: The acceptance criterion is now made more strict, in our case this means increasing the number of required generations by a factor of 10 to 1000. All previously successful parameter vectors v_i are now re-used but their values are perturbed. We achieved good results — measured by rapid yield of acceptable solutions — by a logarithmic perturbation. Thereby, each individual parameter in the given parameter vector v_i is perturbed by multiplication by of division with a random number, sampled from a normal distribution. We again demand n_{sol} successful solutions to the stricter criterion. By this procedure, the parameter space around previously accepted, but likely suboptimal, solutions is explored. Starting from $u_+ \geq 10^{-4}$ and $h_+ \geq 10^{-4}$ and using several tiers of the IPS procedure, we are able to obtain solutions that sustain 10^5 generations of stability, a consequence of an intricate balance of the methylation and de-methylation reactions shown in Fig. 1D, main text. In two separate samplings where we fix $u_+ = 10^{-3}$, $h_+ = 10^{-3}$ and $u_+ = 10^{-2}$, $h_+ = 10^{-2}$, respectively, we are able to achieve results even for 10^6 generations (as shown in Fig. 3, main text).

Parameter sampling in the spatial model

The parameter sampling in the spatial model is carried out analogously. However, the heterogeneous state where the CpG island region is in the U-state while the surroundings are in the M-state requires additional testing. In this particular configuration, a stronger test of the U stability within the CpG island would be to remove the replication step (as described above for the isalated CpG island). However, removing the effect of replication would remove the strain on the M-state in the surroundings of the CpG island. We therefore perform a different test here: After each generation i (immediately before the subsequent replication step) we record the densities, e.g. $m(i)$. We then compute the differences of the current and the previous generation, e.g. $m(i) - m(i-1)$. Repeating this procedure for all generations and collecting the resulting differences, we ask if there is a significant drift of the densities by performing a t-test on the distributions of differences. A significant deviation from zero would indicate a slowly drifting state, i.e. indicate a slow decay of the average methylation density. We only consider motifs to be successful when this test indicates no significant drift.

3 Robustness Analysis

We describe the variations causes by changes in several parameters: The duration of a generation in units of reaction attempts, the number of generations required for accepting a particular model as being able to sustain bistability, and the effect of removing links in the collaborative model, i.e. the quest for *minimal model* of bistability in DNA methylation.

Dependence on the number of generations

The collaborative motif has a finite probability to transition between the M and U state at any given generation, especially after the abrupt replication events. We explore this probability, by resampling from successful motifs (Fig. S4). We yields an exponential distribution of lifetimes of the resulting simulations. Such a distribution is indicative of a Markovian process, where the probability of breakdown during any given generation is independent of the number of generations sustained before that time. Note that this is different in the case of the standard motif, where breakdown is a gradual process and reaching the limit where the dominant state, say u, drops below one of the other two states, say m would be dependent on the history of the state evolutions.

Dependence on system size

We have also explored the dependence on system size. In our default simulations, we have used 80 CpG sites as the standard length of the CpG island. This value is on the lower end of the distributions of CpG island lengths found experimentally [Takai and Jones, 2002]. We have therefore considered also substantially larger lengths of CpG islands. Our general finding is that bistability of such larger systems is much higher. This is due to the reduction of the variance of densities, i.e. the contribution to the relative densities caused by the stochastic fluctuations of the transitions between u, h and m states are reduced. Our results for bistability can be seen as applying even more strictly for larger system sizes. Note that no additional stability can be gained for the standard model where the decay rate of densities is independent of system size.

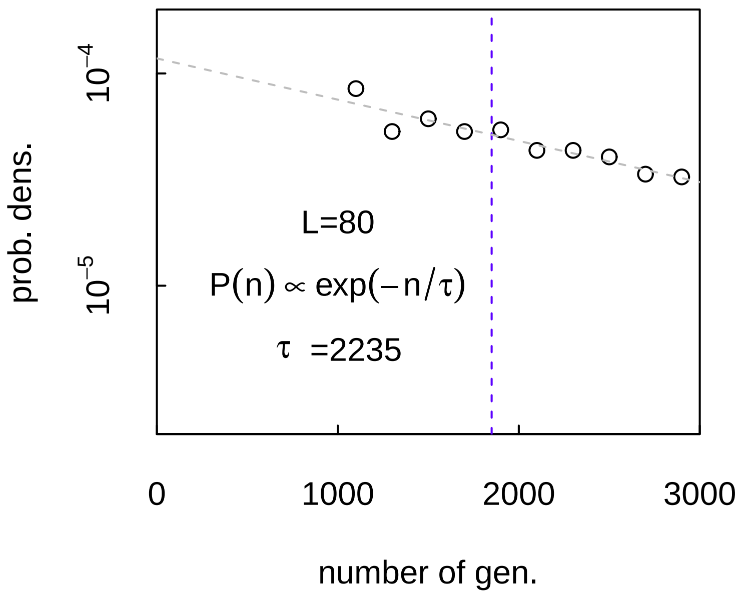

Figure S4: **Probability distribution of lifetimes.** For a number of successful motifs for the standard length of $L = 80$ sites the corresponding parameters were re-sampled and the number of generations before breakdown recorded. The gray dashed line is an exponential fit to the distribution function, τ is the typical decay duration and the blue dashed line indicates the distribution mean of all sampled data beyond 1000 generations.

Systematic link removal and the minimal model

By sequentially removing links from the collaborative motif (Fig. 1D, main text) we obtain a mimimal motif, characterized by the fact that any further link removal will destroy bistability (Fig. S5). Interestingly, in this motif no collaborative de-methylation reactions are required, yet removal of any of the three collaborative methylation reactions is prohibited. De-methylation is required for the maintenance of the U-state, a state not subject to the strong perturbations caused by replication. Therefore, its maintenance can be secured by elevated levels of non-collaborative de-methylation, namely h_- and m_- alone (Fig. S5A). Conversely, methylation reactions are required to maintain the M-state, a state that has to be resistant towards the abrupt, and radical, replication reactions. We find that this can only be warranted by sizable collaborative methylation reactions (Fig. S5B), i.e. all three collaborative methylation reactions h_+^h, h_+^m and u_+^m are needed. Interestingly, for this minimal motif, the M-state is characterized by an incomplete polarization (away from $m = 1$, see Fig. S5D). Sizable densities of both h and u result, more compatible with recent experiments by bi-sulfite sequencing [Laird et al., 2004] than the optimal motif (compare Fig. 2D and E, main text). The U-state is again characterized by all but complete polarization

towards $u = 1$.

We have also explored intermediate motifs where only one of the advantageous collaborative de-methylation links, i.e. either h_-^u or m_-^u, is present. The corresponding results for the distributions of the active parameters and methylation densities are similar to those for the full motif. This supports the conclusion that any form of collaborative de-methylation strengthens polarization of both the M and U-state.

References

Antequera, 2007. Antequera, F. (2007). CpG Islands and DNA Methylation. Wiley Online Library.

Laird et al., 2004. Laird, C. D., Pleasant, N. D., Clark, A. D., Sneeden, J. L., Hassan, K. M. A., Manley, N. C., Vary, J. C., Morgan, T., Hansen, R. S. and Stöger, R. (2004). Hairpin-bisulfite PCR: assessing epigenetic methylation patterns on complementary strands of individual DNA molecules. Proceedings of the National Academy of Sciences of the United States of America *101*, 204–9.

Takai and Jones, 2002. Takai, D. and Jones, P. A. (2002). Comprehensive analysis of CpG islands in human chromosomes 21 and 22. Proceedings of the National Academy of Sciences of the United States of America *99*, 3740–3745.

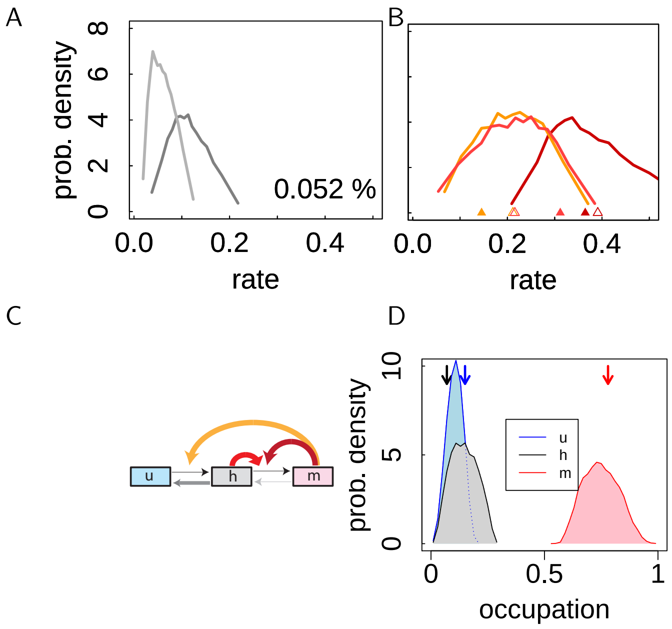

Figure S5: Minimal motif. (A), Probability distribution corresponding to stable realizations for the minimal motif for non-collaborative parameters h_- and m_- where u_+ and h_+ are sampled in $[0.0001, 0.0016]$. Probability listed in (A) is the likelihood of obtaining a bistable solution from the parameter sampling. (B), Probability distributions for collaborative methylation parameters h_+^h, u_+^m and h_+^m. Open (filled) triangles in b and c indicate mean of rates (mean rates that fit the experiment of [Laird et al., 2004]). (C), Schematic of the minimal motif with reactions colored corresponding to the curves in the panels A,B and thicknesses of arrows corresponding to the approximate rates of reactions. (D), Probability distribution of average unmethylated (u), hemi-methylated (h) and fully-methylated (m) CpG sites (right column) in M-state sampled across accepted parameter sets. Vertical arrows (right) indicate the experimentally measured $u = 0.15$, $h = 0.07$, and $m = 0.78$ [Laird et al., 2004].
